# Has Selection for Improved Agronomic Traits Made Reed Canarygrass Invasive?

**DOI:** 10.1371/journal.pone.0025757

**Published:** 2011-10-03

**Authors:** Andrew R. Jakubowski, Michael D. Casler, Randall D. Jackson

**Affiliations:** 1 Department of Agronomy, University of Wisconsin–Madison, Madison, Wisconsin, United States of America; 2 United States Department of Agriculture–Agricultural Research Service, U.S. Dairy Forage Research Center, Madison, Wisconsin, United States of America; 3 United States Department of Energy–Great Lakes Bioenergy Research Center, University of Wisconsin–Madison, Madison, Wisconsin, United States of America; University College London, United Kingdom

## Abstract

Plant breeders have played an essential role in improving agricultural crops, and their efforts will be critical to meet the increasing demand for cellulosic bioenergy feedstocks. However, a major concern is the potential development of novel invasive species that result from breeders' efforts to improve agronomic traits in a crop. We use reed canarygrass as a case study to evaluate the potential of plant breeding to give rise to invasive species. Reed canarygrass has been improved by breeders for use as a forage crop, but it is unclear whether breeding efforts have given rise to more vigorous populations of the species. We evaluated cultivars, European wild, and North American invader populations in upland and wetland environments to identify differences in vigor between the groups of populations. While cultivars were among the most vigorous populations in an agricultural environment (upland soils with nitrogen addition), there were no differences in above- or below-ground production between any populations in wetland environments. These results suggest that breeding has only marginally increased vigor in upland environments and that these gains are not maintained in wetland environments. Breeding focuses on selection for improvements of a specific target population of environments, and stability across a wide range of environments has proved elusive for even the most intensively bred crops. We conclude that breeding efforts are not responsible for wetland invasion by reed canarygrass and offer guidelines that will help reduce the possibility of breeding programs releasing cultivars that will become invasive.

## Introduction

Plant breeding has played a critical role in increasing food and fiber production throughout the world [1]. Continued crop improvement will be necessary to increase food production and meet the increasing demand of fiber for animal feed, manufacturing and as a cellulosic bioenergy feedstock [2], [3], [4]. However, a major concern is the potential development of novel invasive species through breeding efforts and the introduction of exotic crops [5], [6], [7]. Many of the traits associated with invasion potential, such as rapid growth rates, plasticity across a range of environments, high yield, and cold and drought tolerance, are targets for improvement in crops [6], [8], [9], [10], [11], [12]. In addition, crops are more likely to become naturalized or invasive because their introduction is likely to occur at a scale that will establish large founder populations with sustained establishment efforts [13].

Recent efforts to assess invasion by crops has focused on potential biofuel feedstock crops by using weed risk assessment modeling [5], [7]. However, risk assessment is difficult when unknown environmental (climate change) and evolutionary (breeding and hybridization) changes interact to facilitate invasion by a species [14]. A complementary approach to weed risk assessment modeling is to evaluate the history of perennial crop breeding to determine whether improvements in agronomic traits have fostered development of invasive species in the past. There is a long history of perennial crop breeding with a particular focus on grasses and legumes for agriculture [15]. Herbaceous perennial crops that have undergone significant selection efforts include *Phalaris arundinacea* (reed canarygrass), *Schedonorus phoenix* (tall fescue), *Schedonorus pratensis* (meadow fescue), *Lolium perenne* (perennial ryegrass), *Dactylis glomerata* (orchardgrass), *Bromus inermis* (smooth bromegrass), *Poa pratensis* (Kentucky bluegrass), *Phleum pratense* (timothy), *Medicago sativa* (alfalfa), *Trifolium pratense* (red clover), and *Trifolium repens* (white clover) [16]. Many of these crops are now ubiquitous in temperate regions across North America and Eurasia. Most have been identified both as beneficial agricultural species and weedy invaders depending on the context [17], [18]. Using reed canarygrass as our case study, we evaluate the history of introduction and breeding efforts of the species to evaluate the risks associated with breeding perennial crops.

Reed canarygrass is a circumboreal cool-season grass native to North America, Europe, and Asia [19]. The species has been planted for forage since the early 19^th^ Century in North America, actively harvested in Europe since at least 1806 [20], and recently identified as a potential cellulosic biofuel feedstock [21], [22], [23]. Because of the importance of the species to the grazing community, active breeding programs have existed since the early 20^th^ Century with a focus on improving yield, quality, and palatability [24]. However, the species is considered one of the most noxious wetland invaders in the northern United States because it displaces native species and forms monocultures in disturbed wetlands [25]. Hypotheses for the development of reed canarygrass invasiveness in North America include; introduced wild European and Asian populations outcompeted native North American populations [26], formerly distinct populations crossed to create populations with increased genetic variability and hybrid vigor [27], and more aggressive cultivars released by plant breeding programs outcompeted or introgressed with wild populations [19]. Many in the invasion science and management community have called for the banning of breeding programs and the sale of seed, although the hypotheses listed above have not been evaluated [25]. For these reasons, reed canarygrass serves as a useful model for evaluating the effects of breeding on the invasiveness of a species.

Here, we address four objectives. First, we evaluate the genetic similarity between European wild populations, cultivars, and North American invaders. Second, we compare the production and fecundity of cultivars, European wild populations, and North American invader populations of reed canarygrass. Third, we determine whether cultivars selected for improved yield in an agricultural setting (upland soils with nitrogen addition) also show improved yield in non-agricultural settings (unfertilized uplands and wetlands). Fourth, we offer guidelines that will help reduce the possibility of breeding programs releasing cultivars that will become invasive.

## Methods

### Experimental Design

In the summer of 2008, we established common gardens testing four groups of reed canarygrass populations. The four groups, each consisting of three populations were North American cultivars, European cultivars, European wild, and North American invader populations (Table 1). The populations chosen within the European groups included at least one population from the Scandinavian and central European refugia suggested by previous research [28]. The seed of eight of the twelve populations was obtained from the USDA-ARS Germplasm Resources Information Network (http://www.ars-grin.gov), while the three North American invader populations were collected from invaded wetlands in Iowa, Wisconsin, and New York, USA. Seed of Bamse, a European cultivar, was obtained through a commercial seed dealer. Plants were grown as plugs from seed in the greenhouse and transplanted into each of the common gardens. Differences in germination and establishment vigor were considered population-specific effects and were not normalized prior to planting in the common gardens. The upland common garden was established at the University of Wisconsin OJ Noer Turfgrass Research Facility in Verona, WI in June 2008. The design was a randomized complete block with split-plot restrictions comparing two nitrogen treatments (no nitrogen addition and 8 g/N/m^2^ applied twice annually in June and September of each year as ammonium nitrate) with 12 replicates of each treatment. Plants were space planted at 1-m intervals, with a 2-m buffer space between nitrogen treatments. Two years of morphological and production data were collected.

**Table 1:** Accession information.

Name	Code	Group Name	Improvement Status	Origin	USDA GRIN PI #	*n*
Bellevue	Be	North American Cultivar	Cultivar	Canada	PI 587092	2
Vantage	Va	North American Cultivar	Cultivar	USA	PI 578794	2
Venture	Ve	North American Cultivar	Cultivar	USA	PI 531089	2
Rensselaer Falls	Rf	North American Invader	Unknown	New York, USA	N/A	5
Little Eau Pleine River	Le	North American Invader	Unknown	Wisconsin, USA	N/A	4
Hendrickson Marsh	Hm	North American Invader	Unknown	Iowa, USA	N/A	5
Bamse	Ba	European Cultivar	Cultivar	Sweden	N/A	2
Nakielska	N	European Cultivar	Cultivar	Poland	PI 272123	2
Lakeside LA	L	European Cultivar	Cultivar	Hungary	PI 587193	2
Uppsala	U	European Wild	Wild	Sweden	PI 235547	5
D-1827	D	European Wild	Wild	Russia	PI 440584	2
Mountain Swiss	Ms	European Wild	Wild	Switzerland	PI 235485	5

The name, origin, and improvement status of the 12 populations included in the study. Sample size (*n*) refers to the number of samples included in the genetic analysis.

A second garden experiment was established during the summer of 2008 in a simulated wetland in a greenhouse at the University of Wisconsin West Madison Agricultural Research Station. The populations were fully randomized within the simulated wetland with six replicates per population. Plugs were planted into 19-L pots of nitrogen-rich homogenized field soil and placed in a pool with 30 cm of standing water. Plants were grown for 122 days before harvest in the wetland garden and 134 days before harvest in the upland garden.

### Genetic analysis

Additional seed of each population was germinated in the greenhouse in 2008. Fresh tissue (0.1 – 0.2 g) was collected approximately three weeks following germination and frozen for future use. Total genomic DNA was obtained using standard methods [29] and normalized to approximately 10 ng/ul. Between two and six samples per population were evaluated.

One hundred and thirty SSR primer pairs developed for use with *Phalaris canariensis* were synthesized and evaluated for amplification and allelic polymorphisms [30]. The forward primer of each pair was synthesized with the universal M13 tail (CACGACGTTGTAAAACGAC) at the 5′ end to facilitate fluorescent labeling [31]. The M13 tail was labeled either with carboxyfluorescein (FAM) or hexachlorofluorescein (HEX) fluorescent tags. In addition, to reduce polyadenylation and improve genotyping, the PIG sequence (GTTTCTT) was appended to the 5′ end of the reverse primer [32]. Of these primer pairs, only 11 yielded polymorphic and easily scored alleles without extraneous peaks (Table S1). Four additional primers were included that were developed from a project identifying conserved primers within the *Poaceae* by downloading 2,340 SSR primers from Maize GDB [33] and finding perfectly conserved primer sequences in the *Sorghum bicolor* (sbi1) genome [34]. Polymerase chain reactions (PCR) were performed in 8 ul total volume using 3.5 ul 1X JumpStart REDTaq ReadyMix (Sigma, St. Louis, MO, USA), 10 ng genomic DNA, 1.25 ul of H2O, 0.5 ul 5 M M13-FAM/HEX primer, 0.5 ul 5 M reverse/0.5 M forward primer, 0.125 ul 5 M betaine (Sigma, St. Louis, MO, USA), and 0.125 ul 50 mg/ml BSA (CHIMERx, Milwaukee, WI, USA). Thermocycling conditions consisted of an initial melting step (94°C for 3 min), followed by 35 cycles of 94°C for 15 s, 54°C for 90 s, and 72°C for 2 min, and an elongation step (72°C for 20 min), followed by an indefinite soak at 4°C. PCR products (2 ul) using different fluorescent labels (i.e., FAM and HEX) were pooled and combined with 15 ul Hi-Di formamide (Applied Biosystems, Foster City, CA, USA) and 0.15 ul of carboxy-X-rhodamine (ROX) custom size standard (Custom MapMarker, BioVentures, Murfreesboro, TN, USA). SSR allele genotyping was performed using an ABI 3730 fluorescent sequencer (POP-6 and a 50-cm array; Applied Biosystems, Foster City, CA, USA). Alleles were scored using GeneMarker Software version 1.7 (SoftGenetics, State College, PA, USA).

Data obtained with the SSR loci were scored in a binary format as presence (1) or absence (0) of bands. A pairwise individual-by-individual Euclidean distance matrix was calculated in GenAlEx 6.4 [35] for the binary data and was used to perform the following analyses. Principal component analysis (PCA) was performed based on the genetic distance of all samples. An analysis of molecular variance (AMOVA) was calculated to test for significance of genetic variation between groups and populations, and to confirm that the North American invader and European wild populations were distinct from the cultivars. Significance was determined by comparing actual values to a null distribution generated from 9,999 permutation of the data.

### Phenotypic analysis

Aboveground biomass production was measured using allometric equations developed from the harvest of additional plants. Unique allometric equations were developed with multiple regression for each year to account for changes in plant growth using canopy height, canopy diameter, and basal perimeter to predict aboveground biomass. Aboveground biomass was clipped in early November of each season, dried at 65°C for four days, and weighed. Canopy height was measured as the distance from the soil surface to the height of the highest leaf. Canopy diameter was measured as the distance between the outermost leaves in the canopy. Basal perimeter was measured as the distance around the outermost tillers of the plant at the soil surface. Because of the strong relationship between these vegetative characteristics and aboveground biomass, we use aboveground biomass production as our estimate of vegetative vigor.

Belowground production allometric equations were developed for the upland plants over a 2-year period in which 85 additional plants were harvested to determine total belowground production. The plants were harvested by digging all soil within approximately 15 cm of the outermost tillers and to a depth of approximately 35 cm. The root mass of each plant was hand washed, dried at 65°C for four days, and weighed. End-of-season aboveground biomass was used as a predictor of belowground production [36], [37]. The roots of all plants in the wetland garden were washed, dried, and weighed.

An index of fecundity of each plant was developed using PCA to integrate values of the number of panicles, average panicle length and average panicle height of each plant into a single value. Because plants did not flower in the upland garden during the first season, the index was calculated using first year data in the wetland garden and second year data in the upland garden. Average panicle length was measured for three panicles on each plant. Average panicle height was measured as the distance from the soil surface to the highest point of three panicles per plant. The number of panicles of each plant was counted in late July in the uplands and prior to final harvest in the wetland. The first principal component generated from this analysis was normalized to a range of zero to one [38]. The positive relationship between each trait and the index was confirmed using Pearson correlation coefficients.

Maximum likelihood mixed-effects models were developed using the NLME package in R 2.10.1 to test the significance of fixed effects. Linear mixed models were compared to a null model using a likelihood ratio test to determine whether fixed effects were significant at p = 0.05 [39]. A likelihood ratio test was used to compare two models for each response variable: group as a fixed effect vs. group and populations nested within group as fixed effects. Group, population, year, and environment (upland with nitrogen addition, upland with no nitrogen addition, and wetland) were treated as fixed effects, while block was treated as a random effect. Year was used as a fixed-effect repeated measure to account for expected differences in morphology between the establishment and persistence years, allowing estimation of the autoregressive correlation structure between years. Variables were log transformed as needed to meet normality assumptions.

## Results

**Figure 1:**
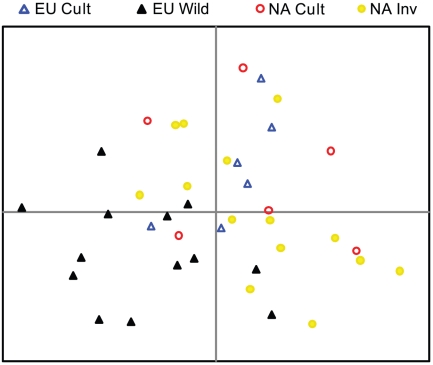
Genetic distance of populations. Principal component analysis based on Euclidean genetic distance of 39 samples from four groups made up of three populations each based on 15 SSR markers.

The PCA based on genetic data from the 39 samples did not identify any completely distinct clusters, and emphasized the diversity represented by both North American and European cultivars (Figure 1). European wild populations were the most distinct from all other groups, but all four groups had some degree of overlap. The first two axes of this PCA accounted for 41% of the total genetic variation. The AMOVA results determined that the populations were significantly different genetically (p<0.0001), accounting for 15% of the total genetic variation (Table 2). The group classification did not explain a significant amount of variation, explaining only 2% of the total genetic variation (p = 0.16). The remaining 84% of variation was present within populations indicating the high level of genetic diversity present within each cultivar and population.

**Table 2:** Analysis of molecular variance (AMOVA) table.

Source of variation	*df*	SS	MS	Variance Component	Percentage of variation	p
Among Groups	3	43.4	14.5	0.2	2%	0.16
Among Populations	8	103.1	12.9	1.5	15%	<0.0001
Within Populations	27	223.7	8.3	8.3	84%	<0.0001
Total	38	370.2		9.9	100%	

Analysis of molecular variance (AMOVA) by group and population based on 15 SSR primers.

The R^2^ value of the allometric equations for predicting aboveground biomass using morphological measurements was 0.81 in year one and 0.89 in year two. The R^2^ value of the equation predicting belowground production using aboveground production was 0.69 across both years.

**Figure 2:**
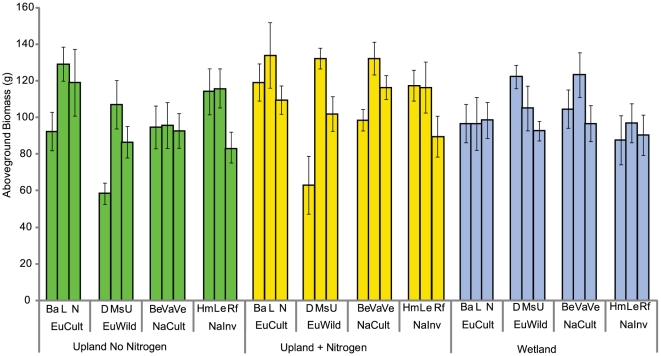
Aboveground production in year one. The aboveground production (Means ± S.E.) of the 12 populations compared across the three environments in year one. The group x environment interaction was significant (p = 0.001). The European wild group was less productive than all other groups in both upland environments (p = 0.002 and p = .01, respectively). There were no significant differences in production in the wetland environment (p = 0.28).

For all response variables, the use of a separate estimate for each population significantly improved models using an estimator for each *a priori* group (p<0.05), suggesting that there was significant variability between the populations within a group. In year one, both group x environment and population x environment interactions were significant (p<0.001 for both, Figure 2). In both upland environments, the European wild group was less productive than all other groups (no nitrogen, p = 0.002 and nitrogen addition, p = 0.01). Neither group nor population were significant in the wetland environment (p = 0.11 and p = 0.36, respectively).

Similar patterns were found for belowground production in the first season. In both upland environments, the European wild group produced significantly less biomass (p<0.001). Neither group nor population were significant predictors of belowground biomass in the wetland environment (p = 0.11, and p = 0.12).

**Figure 3:**
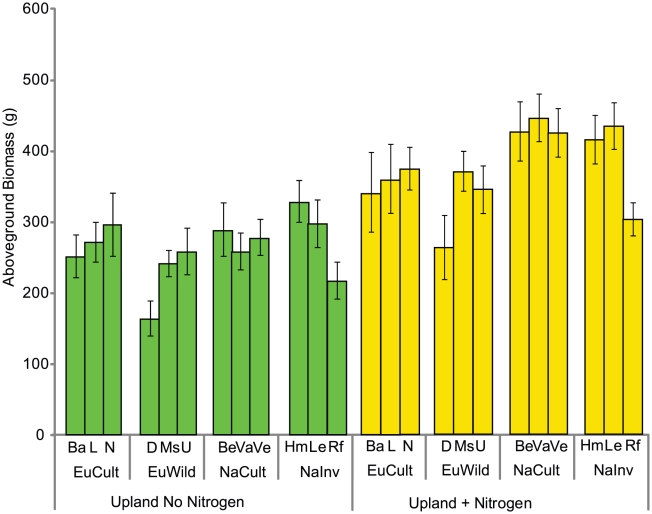
Aboveground production in year two. Aboveground production (Means ± S.E.) of the twelve populations compared across the two upland environments in year two. The group x year interaction was significant (p<0.0001), as was the environment x year interaction (p<0.001), but the group x environment interaction was not significant (p = 0.72). The European wild group was significantly different from all other groups (p<0.001).

We were unable to maintain populations in the simulated wetland under realistic conditions for two seasons because of the inability to simulate overwintering and a lack of space due to plant size, so we were unable to test group x year interactions in this environment. However, upland experiments were evaluated for two growing seasons. Both group x year and population x year interactions were significant (p<0.001), suggesting differences in vigor in the establishment and persistence years (Figure 3). The nutrient treatment x year interaction was also significant, likely due to an increased nitrogen limitation in unfertilized plots in year two (p<0.001). The group and population x upland nutrient treatment interaction were not significant (p = 0.33 and p = 0.72), indicating that groups and populations were not responding differently to nitrogen addition. On average, the addition of nitrogen increased production by 115.3 (±13.0) g/plant. In both environments, the European wild group was significantly less productive than all other groups (p<0.001). Because of the strong correlation between aboveground and belowground production in the upland treatments, differences in belowground production between populations in year two are similar to aboveground differences and are not reported.

**Figure 4:**
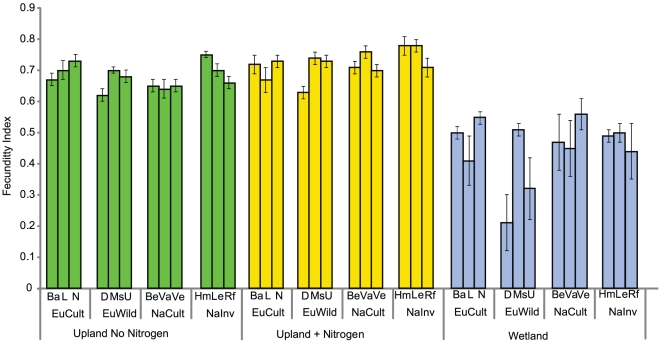
Fecundity of populations. The fecundity index (Means ± S.E.) compared across the different environments. The population x environment interaction was significant (p = 0.01). In uplands without nitrogen addition, the North American cultivar group was significantly less fecund than all other populations (p = 0.01). In uplands with nitrogen addition, the invader group was significantly more fecund than all other groups (p = 0.007). In wetlands, the European wild group was significantly less fecund than all other groups (p = 0.005).

The first principal component of the fecundity index extracted 60% of the variation of the three traits used. The first principal component was positively correlated with each of the three traits used to generate it (p<0.001) and had an average Pearson correlation coefficient with the three traits of 0.74 (±0.20 S.D.). Both the group and population x environment interactions were significant for the fecundity index (p = 0.009 and p = 0.01, Figure 4). In uplands with nitrogen addition, the North American invader group was significantly more fecund than all other groups (p = 0.007). In the wetland environment, the European wild group was significantly less fecund than all other groups (p = 0.005). The fecundity index value was correlated with end of season production (year one in wetland, year two in upland environments) with a Pearson correlation coefficient of 0.65.

## Discussion

### Understanding the origins of reed canarygrass invasion

Breeding efforts do not appear to be the primary reason for invasion by reed canarygrass, but our data does not completely rule out the possibility. The genetic data offers conflicting evidence regarding the genetic origin of invasive populations. The PCA clusters the three European wild populations as somewhat distinct from cultivars, but among-group variability was not significant in the AMOVA. The invasive populations overlap with both groups of cultivars and have only minor overlap with the European wild populations. Two hypotheses could explain these results. First, cultivars are thought to be derived from wild European germplasm and should represent significantly less diversity than wild European germplasm due to selection and genetic bottlenecks [40]. If this hypothesis is correct, the three European wild populations used in this study are not representative of the total diversity of the species in Europe. Alternatively, our results could be accurate in suggesting that invasive populations in North America are more similar to cultivars than to wild European germplasm. Introductions by early European settlers of agronomic varieties were likely the founding populations of present-day invasive populations in North America. The progeny of these early introductions may make up the majority of invasive populations in North America, regardless of whether breeding efforts made the species more invasive. Additional sampling of European and North American populations is necessary to evaluate these hypotheses further.

Previous research suggested that increased genetic variability in North American reed canarygrass populations resulted in increased vegetative vigor when compared to European wild populations [27]. Our results do not support this hypothesis of higher vigor and phenotypic variability in North American invader populations over European wild populations. There was significant phenotypic variability among populations within each continent, suggesting that a plant's provenance is not a good predictor of its vigor. One of the European wild populations, Mountain Swiss, collected in an Alpine meadow in Switzerland was as productive and fecund as the most vigorous and fecund population in all environments in year one. The European wild population D-1827, collected in Russia, was consistently the least productive and fecund in upland environments, was as productive as all other populations in the wetland environment, but was significantly less fecund in the wetland environment. However, the fecundity index from the wetland environment may not be appropriate because plants did not undergo vernalization, which is generally necessary for flowering in the field [41]. Our results suggest that the least vigorous European populations may have been eliminated in North America following their introduction due to evolutionary pressure, but our inference here is limited by our small sampling of populations from both continents. Alternatively, the planting of early introductions and cultivars could have made the species more common in North America, even though improvement of traits via breeding have had a minimal effect on the invasiveness of the species. Additional support for this hypothesis would come from a genetic study of a large number of European samples, cultivars, and North American invasive populations to determine the genetic similarity of European and North American populations. The fecundity of cultivars and North American invader population was no higher than some European wild populations. While increased fecundity may have been a goal of early breeding programs [42], the goal of improving forage quality may have unintentionally selected against highly fecund individuals as increased flowering can reduce forage quality [43].

Early cultivars of reed canarygrass frequently exhibited issues of low palatability and resulted in suppressed intake and weight gain by ruminant livestock due to the presence of indole alkaloids [43]. As a result, breeding programs focused on improving palatability by altering alkaloid profiles during the past 30+ years [40]. This selection for improved palatability likely has reduced the fitness of cultivars by making them more susceptible to insect herbivory [44]. Further, the selection for improved palatability has limited the ability to select for increased yield by constraining the available germplasm from which selections can be made. A recent evaluation of 72 wild accessions collected in North America and eight cultivars found that 39 of the wild accessions ranked higher in biomass yield than all eight cultivars [45]. A similar limitation is likely to occur in cellulosic biomass crops due to efforts to alter lignin and cellulose production to improve the efficiency of conversion to ethanol [46].

The hypothesis that breeding for improved agronomic traits is responsible for creating invasive populations of reed canarygrass is not supported by our results. While cultivars are consistently among the highest yielding populations in the environment for which they were selected (uplands with nitrogen addition), these populations are not significantly more productive than the highest producing European wild population in uplands, and there were no differences in above- or below-ground production in the wetland environment. This suggests that breeding efforts in reed canarygrass have had little effect on biomass production in upland environments and no significant effect on production in wetland environments. This is not surprising, as it has proven difficult in many crops to achieve improvements that are consistent across a wide range of environments [47], [48] If breeding were the primary cause of the invasive traits of the species, we would expect cultivars and North American invasive populations to be far more productive than all European wild populations in wetland environments. While possible, it is unlikely that marginal increases in production or fecundity are responsible for a species becoming invasive. In our study, the most fecund population in wetlands, *Vantage*, a North American cultivar, was 10% more fecund than the most fecund European wild population. Previous research comparing seed yield of eight cultivars, 14 breeding populations, and 53 European wild populations found wide variation among populations within each group, with four wild populations more fecund than the most fecund cultivar, *Vantage*. The wild population with the highest seed yield had a seed production index 17% higher than Vantage, lending further support to the hypothesis that breeding has not led to the development of levels of seed production not already found in wild populations [49]. While reed canarygrass is considered an extremely plastic species due to its ability to flourish in upland and lowland environments [50], [51], our data suggests that improvements from breeding have not been maintained across all environments. Breeding and the intentional planting of cultivars may have resulted in a higher abundance of the species on the landscape, but breeding does not appear to be responsible for biomass production and fecundity levels not already found in wild European populations.

Additional research is necessary to understand how this native species has become invasive, but previous research has concluded that the species is successful in eutrophic, highly disturbed wetlands [52], [53], [54]. This niche has expanded greatly in North America following the expansion and intensification of agriculture during the 20^th^ century [55]. Rather than changes in aggressiveness of the species among European populations being responsible for invasion, an increase in the ideal environment of the species may be the driver of invasion by reed canarygrass [56].

### Applying knowledge to the risks of perennial breeding

Using reed canarygrass as an analogue for the effects of breeding on perennial crops is most appropriate for species with a similar life history (e.g. switchgrass); however, our study offers useful insights to minimize the risks associated with breeding perennial crops. The risks of invasion by crops in the near future will be dominated by the introduction of novel bioenergy crops, a legitimate concern given a recent estimate of $26.4 billion for the costs of management and lost production due to invasive weeds in the US agricultural economy [57]. The simplest response to this concern is to restrict the use of any exotic or improved crops deemed potentially invasive as a precautionary measure. While developing systems to screen out the most likely invaders will be important [7], preventing the use of all possible improved crops eliminates the possibility of the many beneficial ecosystem services that accompany crop improvements and may accompany a shift to a bioenergy economy [58], [59].

There are alternatives to allowing all species to be planted or highly restricting any potential invaders. In reed canarygrass, improvements in yield were only present in upland environments, and were not maintained in wetland environments. Breeding often selects improvements for a very specific target population of environments, and stability across a wide range of environments has proved elusive for even the most highly bred crops [48]. Selecting cultivars for specific environments or requiring specific management (i.e. high nitrogen environments or cultivation) reduces the probability that a crop will escape cultivation. Breeders generally strive for cultivar stability across a range of environments, as these cultivars are more marketable and simplify selection for a producer [47]. In contrast to typical cultivar development, a range of cultivars that are each designed for a specific environment may be preferable for biofuel feedstock crops. In addition, the evaluation of crops in environments in which they have the potential to be invasive may be appropriate to select against the populations that are highly productive in these environments. This will require collaboration between breeders and weed scientists to determine which environments are most at risk.

Another safeguard to reduce the chance of creating a novel invader is to select for reduced fecundity of perennial crops. Efforts to select for slowed maturity and decreased flowering to improve forage quality have been successful in several perennial pasture species [60], [61]. In addition, research to reduce or eliminate flowering by altering the genetic mechanisms involved in the flowering pathway has shown promise [62], [63]. This technique not only reduces the fecundity of the crop but can also increase biomass production due to a reallocation of resources. Selecting for reduced fecundity in biomass crops has the potential to reduce the risks of introgression of improved genes or traits into native populations and the escape of improved varieties into undesirable environments.

### Conclusion

Our reed canarygrass study suggests that breeding efforts do not appear to be the source for the invasive traits of the species. Cultivars were among the most productive populations in the environment for which they were selected (uplands with nitrogen addition), but were not more productive or fecund than all wild populations in the environment in which they are considered invasive. While early introductions of the species for agriculture likely acted as the initial founding populations prior to invasion, there do not appear to be major differences in biomass production or fecundity between modern cultivars and wild populations in wetland environments. Additional research is required to evaluate the risks associated with the introduction of novel crops and cultivars. To achieve this goal, an understanding of the interaction of landscape changes and human-influenced evolutionary change (either direct as in breeding or indirect through the introduction of exotic species) is necessary. While every crop is different, the study of historical perennial crop breeding can help to identify issues and solutions before novel crops or cultivars are released on the landscape.

## Supporting Information

Table S1 SSR primer sequences. Sequences of the 15 SSR primers used in the genetic analysis.
(XLS)
